# Pneumotomy

**Published:** 1890-05-03

**Authors:** 


### PNEUMOTOMY.

This is perhaps the latest achievement of visceral surgery, which itself owes its great development of late to Lister's antiseptic and aseptic procedure. Dr. Ramsay, of St. Cloud, U.S., reports four cases, three of which have been perfectly successful, one for gangrene, consequent on pneumonia, in which little more than an inch of the seventh rib was removed in the axillary line and the cavity opened and drained. The patient was discharged cured in six weeks and remains in good health and at work. We reported some time ago a similar case from Italy of gangrene supervening on malarial pneumonia equally successful. Dr. Ramsay's next case was one of abscess following a gun-shot wound in which the patient made a good recovery after excision of portions of the third, fourth, fifth, sixth, and seventh ribs; and his third successful one was that of an abscess occurring after pneumonia with excision of parts of the third, fourth and fifth ribs in the axillary line. His unsuccessful case was one for multiple abscesses following measles in a member of a tuberculous family, and probably himself already tuberculous. In this, evidently unsuitable case, death occurred after two months from septicæmia. A daily paper reports the removal (?) of the lower portion of a tuberculosis lung by Professor Tillmann, of Leipzic with success thus far, but since tubercolosis rarely commences at the base, it was more probably another example of the evacuation of an abscess consequent on pneumonia.

